# A Randomized Crossover Comparison between Team-Based Learning and Lecture Format on Long-Term Learning Outcomes

**DOI:** 10.3390/pharmacy6030081

**Published:** 2018-08-04

**Authors:** Barry E. Bleske, Tami L. Remington, Trisha D. Wells, Kristin C. Klein, Jeffrey M. Tingen, Michael P. Dorsch

**Affiliations:** 1 Department of Pharmacy Practice and Administrative Sciences, College of Pharmacy, University of New Mexico, Albuquerque, NM 87131, USA; 2 Department of Clinical Pharmacy, College of Pharmacy, University of Michigan, Ann Arbor, MI 48109, USA; remingtn@med.umich.edu (T.L.R.); tdwells@med.umich.edu (T.D.W.); kriklein@med.umich.edu (K.C.K.); mdorsch@med.umich.edu (M.P.D.); 3 Department of Family Medicine, University of Virginia Health System, Charlottesville, VA 22904, USA; JMT3BQ@hscmail.mcc.virginia.edu; 4 Department of Clinical and Social Administrative Sciences, College of Pharmacy, University of Michigan, Ann Arbor, MI 48109, USA; 5 Department of Pharmacy Services, University of Michigan Health System, Ann Arbor, MI 48109, USA; 6 Department of Pharmacy Services, University of Virginia Health System, Charlottesville, VA 22904, USA

**Keywords:** active learning, team-based learning, outcomes, lecture

## Abstract

There are limited data evaluating the effectiveness of different teaching pedagogies to maintain gains in learning achieved over the short term. The purpose of this study is to compare long-term learning outcomes between two different teaching pedagogies, team-based learning (TBL) and lecture. Within a therapeutic elective course a randomized crossover study was conducted with 30 students divided into two sections. Each section was taught six therapeutic topics (three TBL and three lecture). Six months following completion of the course, 47 assessment questions (application and recall multiple-choice questions) were re-administered to 16 students from the class with no prior announcement of the assessment. The results showed no significant difference in long-term assessment scores between TBL and lecture formats (67 ± 14% vs. 63 ± 16%, *p* = 0.2, respectively). In addition, there was a significant (*p* < 0.0001) and similar decline in short-term gains for TBL (90 ± 9% vs. 67 ± 14%) and lecture (86 ± 11% vs. 63 ± 16%) in assessment scores. In conclusion, there was no advantage gained by employing an active-learning pedagogy when assessing multiple-choice questions six months following end of a therapeutics course in a limited sample size. Neither pedagogy was able to maintain short-term gains in learning outcomes as assessed by multiple-choice questions.

## 1. Introduction

There has been a growing trend for pharmacy curricula to incorporate active-learning pedagogies and approaches (e.g., team-based learning, flipped class room) to promote higher order learning. Educational research in non-pharmacy disciplines and within pharmacy have shown that active-learning pedagogies may enhance student learning and may lead to neutral or improved short-term outcomes compared to lecture-based approaches [1,2,3,4,5,6,7,8,9,10,11,12]. In fact, we have previously published results from a randomized crossover trial comparing learning outcomes between team-based learning (TBL) pedagogy to more traditional punctuated lecture pedagogy [13]. We observed significantly higher overall examination scores for students taught using TBL using application and recall type questions which is consistent with previous studies that looked at short-term outcomes employing other less rigorous type of study designs [4,5,6,7,8,9,10,11,12]. Pharmacy students need to not only recall facts but more importantly need to be able to apply their knowledge to the patient care setting. Given today’s environment where facts are only a click away, it is most important that students are assessed on their ability to apply their knowledge to different clinical scenarios. In our previous study, we felt it was essential to design and assess not only recall but application type questions. In addition to evaluating short-term outcomes, another objective for our study was to compare TBL to lecture on long-term learning outcomes. We believed this was critical given the lack of data regarding comparison of long-term outcomes between the two pedagogies, especially in a randomized crossover designed trial. Arguably, it is the gain in long-term learning that is of most concern and to that end understanding the best approach in achieving this learning is paramount. The time frame for evaluating long-term learning is at best empirical; our goal in part was to assess after approximately a two semester time frame. For a professional program such as pharmacy the ability to recall and apply concepts learned at different time periods is essential for patient care and safety. This report is part of the process in helping to understand how effective different teaching pedagogies are on long-term learning. The report below describes the findings on learning outcomes six months following the end of a therapeutics elective course.

## 2. Materials and Methods

An advanced elective therapeutics course was prospectively designed to compare TBL to lecture pedagogy in a randomized crossover model. A complete description has been previously presented [13]. Briefly, students (28 second year professional students (P2) and 2 third year professional students (P3)) were randomized (random numbers table) into either TBL or lecture based pedagogies. The students were well versed and experienced with TBL pedagogy and had completed two prior 16-week TBL-based therapeutic courses as standard part of the curriculum. We employed standard TBL method including a readiness assurance process (individual and team readiness assurance tests). In addition, the “4S” (significant problem, same problem, specific choice, simultaneous reporting) approach was used during the recitation period (significant problem, same problem, specific choice, simultaneous reporting). The “4S” for TBL is a systematic approach that provides an interactive and accountable environment that allows students working in small groups to work on activities requiring synthesis and application of new knowledge based on their pre-work. In regard to the lecture-based pedagogy faculty members could employ either a traditional or a punctuated (embedded cases or use active-learning techniques such as think-share-pair) lecture style. The 6 faculty members in the course had taught for an average of 17 years (range 3–25 years). Each faculty taught both TBL and lecture formats. Students were in each pedagogy for a 3-week topic sequence, tested, and then crossed over to the opposite pedagogy for another 3-week topic sequence and tested. Six months after the completion of the course the class was reconvened. The purpose of reconvening the course was not revealed to students in advance and was not mandatory. The two multiple-choice examinations that were initially given during the course were re-administered. There were a total of 47 multiple-choice questions, 23 application based and 24 recall based questions. In comparing items in the TBL or lecture classes, a paired *t*-test was used to compare their means, *p* < 0.05 was considered significant. The study was approved by the institution’s Health Sciences and Behavioral Sciences review board.

## 3. Results

### Topic Sequence and Test Scores

A total of 16/30 students took the post-course assessment. There were 8 students from the group randomized first to TBL pedagogy and 8 from the group initially randomized to lecture. For the 16 students that did participate in the study, demographics were similar to the 14 students who did not participate (66% and 68% female, respectively). Both groups of students averaged a B grade in the course, although the 16 students that did participate had overall higher course score (88 ± 5%) than the students who did not participate (84 ± 4%, *p* = 0.048). The topic sequence for the course is shown in Table 1. Test scores (% correct) following 6 months after the completion of the course for application, recall, and combined (recall and application) examination questions for each pedagogy are shown in Table 2. No significant differences in examination score were seen between the two teaching approaches.

When evaluating short-term exam scores (exams administered during course sequence) to long-term exam scores (6 months following the end of the course) for each respective pedagogies, there was a significant decline in scores in all areas (Table 3) [13]. The changes were similar for TBL and lecture pedagogies. As mentioned, our previous study showed improved short-term outcomes for TBL. In this current cohort of students, similar findings for short term outcomes were seen (Table 3) with higher numerical scores for the TBL pedagogy; however these higher scores were not statistically different between TBL and lecture (*p* > 0.05).

## 4. Discussion

In one of the only studies employing a randomized, crossover design to evaluate long-term learning outcomes between two different teaching pedagogies, no significant difference in test scores was observed six months following completion of a therapeutics course. For both teaching pedagogies, there was significant and similar decline in scores six months following course completion as compared to in-class scores. Any gain in knowledge or application skills did not appear to persist over time for either pedagogy.

Several studies evaluating short-term learning outcomes between TBL and other teaching pedagogies show similar or improved outcomes with TBL format [4,5,6,7,8,9,10,11,12,13]. However, few investigations examine effect of pedagogy on persistence of learning. In a study by Farland et al., TBL pedagogy was used for 17% of the topics in a therapeutics-based course and they found no difference in either short-term or long-term outcomes between the two pedagogies [9]. They also observed lack of effect on persistence of learning five months following the end of the course (TBL short term 81.84 ± 8.19%; didactic lecture short term 80.50 ± 7.10%, *p* = 0.369; TBL long term 63.65 ± 10.14%, didactic lecture long term 65.43 ± 10.11%, *p* = 0.419). Other studies employing cohort-type designs examining long-term outcomes similarly demonstrated significant deterioration of knowledge over time for both TBL and lecture [11,12]. One of these studies showed no difference in knowledge retention between the two pedagogies. The other study showed improved knowledge retention after 17 months with lecture-based learning (exam scores: 62.9 ± 19.3% vs. 54.9 ± 15.7%, *p* = 0.001).

Our findings and findings from previous studies highlight not only the need to assess long-term learning outcomes but also the need to develop approaches to improving long-term outcomes (or maintain short-term gains in learning). In part, due to accreditation standards (e.g., Accreditation Council for Pharmacy Education, findings of short-term learning gains and student perception on learning, active-learning methods are being incorporated into many curricula. Moves towards “flipped classrooms” and other active or technology driven pedagogies have out-paced broad assessment of these approaches. While there is no evidence that relying on students for initial exposure to content (flipped classroom) compromises their learning, the available evidence does not overwhelm and may not support the large effort/cost associated with moving from traditional instruction to active learning in general or TBL in particular.

Perhaps an important educational question to ask is how to maintain gains in learning over the long term and how to document these gains in a meaningful way. Active-learning pedagogies may have advantages over traditional lecture. In particular, they may enhance team skills, communication skills and clinical reasoning skills through engagement with higher order application exercises. These learning outcomes are largely unassessed, in large part due to unavailability of validated tools to assess them, or difficulty isolating impact of a pedagogy from other aspects in a health professions curriculum. Perhaps capstone type assessments at multiple time points throughout the curriculum may help students maintain short-term gains. Another consideration may be to evaluate long-term gain by evaluating clinical reasoning skills versus retention of knowledge.

The strength of this study lies in the study design (randomized crossover trial) and evaluation of both recall and application type questions. Our results significantly add to the very limited data published in this area, based on studies with less rigorous study designs. While we observed no difference in persistence of learning, our study was limited by low sample size and higher variability in scores following long-term assessment vs. short-term assessment. Other limitations of the study include the fact that only 16 students out of 30 were evaluated on long-term outcomes. The study did not target specific students or group of students, the date picked for re-testing was random and based on the instructor’s schedule. There was no intention to pick specific dates with specific students targeted for participation. Overall, the students in the course tended to be high-performing students (88% average). It is not known if differences in outcomes would be different for lower-performing students. Finally, there were only 47 questions assessed, which in addition to only 16 students evaluated limited our power (~20%). However, trends in the data are clear. Although we evaluated and measured outcomes based on application type multiple-choice questions, other forms of assessment including short answer, essay, or an objective structured clinical examination would be of additional value. These type of assessments may have led to different results.

## 5. Conclusions

This study demonstrated that there was no difference in long-term learning outcomes between TBL and lecture format when evaluating multiple-choice questions after a six-month period with limited sample size. In addition, and importantly, there was significant decline in learning outcomes over time for both pedagogies. Additional exploration is needed to determine not only how to better maintain short-term gains in learning outcomes, but perhaps how to better measure long-term learning outcomes.

## Figures and Tables

**Table 1:** Topic and examination schedule for team-based learning and lecture groups.

Session Weeks	Topics and Examinations
1	Heart Failure and Co-Morbidities
2	Designer Drugs of Abuse
3	Kawasaki Disease
4	Exam
5	*Crossover Pedagogies*: Sexually Transmitted Diseases
6	Obesity Drugs
7	Restless Leg Syndrome
8	Exam

**Table 2:** Long-Term Exam Scores (mean and standard deviation).

Question Type	TBL	Lecture	*p* Value
Application (N = 23)	65 ± 17%	59 ± 16%	0.3
Recall (N = 24)	70 ± 11%	67 ± 15%	0.5
Application and Recall (N = 47)	67 ± 14%	63 ± 16%	0.2

**Table 3:** Short-Term (ST) and Long-Term (LT) Exam Scores (mean and standard deviation).

Question Type	TBL ST	TBL LT	*p* Value	Lecture ST	Lecture LT	*p* Value
Application (N = 23)	91 ± 9%	65 ± 17%	<0.0001	85 ± 12%	59 ± 16%	<0.0001
Recall (N = 24)	90 ± 9%	70 ± 11%	<0.0001	88 ± 9%	67 ± 15%	<0.0001
Application and Recall (N = 47)	90 ± 9%	67 ± 14%	<0.0001	86 ± 11%	63 ± 16%	<0.0001
